# Transmetalation in Surface-Confined Single-Layer Organometallic Networks with Alkynyl–Metal–Alkynyl Linkages

**DOI:** 10.1021/acsnano.4c02263

**Published:** 2024-07-23

**Authors:** Wenchao Zhao, Felix Haag, Ignacio Piquero-Zulaica, Zakaria M. Abd El-Fattah, Prashanth Pendem, Pablo Vezzoni Vicente, Yi-Qi Zhang, Nan Cao, Ari Paavo Seitsonen, Francesco Allegretti, Biao Yang, Johannes V. Barth

**Affiliations:** † Physics Department E20, TUM School of Natural Sciences, Technical University of Munich, James Franck Straße 1, Garching 85748, Germany; ‡ Physics Department, Faculty of Science, Al-Azhar University, Nasr City, Cairo 11884, Egypt; § Physics Department, Faculty of Science, Galala University, New Galala City, Suez 43511, Egypt; ∥ Institute of Physics, Chinese Academy of Sciences, Beijing 100190, China; ⊥ Département de Chemie, École Normale Supérieure, 24 rue Lhomond, Paris F-75005, France; # Institute of Functional Nano and Soft Materials (FUNSOM), Jiangsu Key Laboratory for Carbon Based Functional Materials and Devices, Soochow University, 199 Ren’ai Road, Suzhou, Jiangsu 215123, China

**Keywords:** organometallic network, transmetalation, alkynyl coupling, scanning probe microscopy, surface chemistry

## Abstract

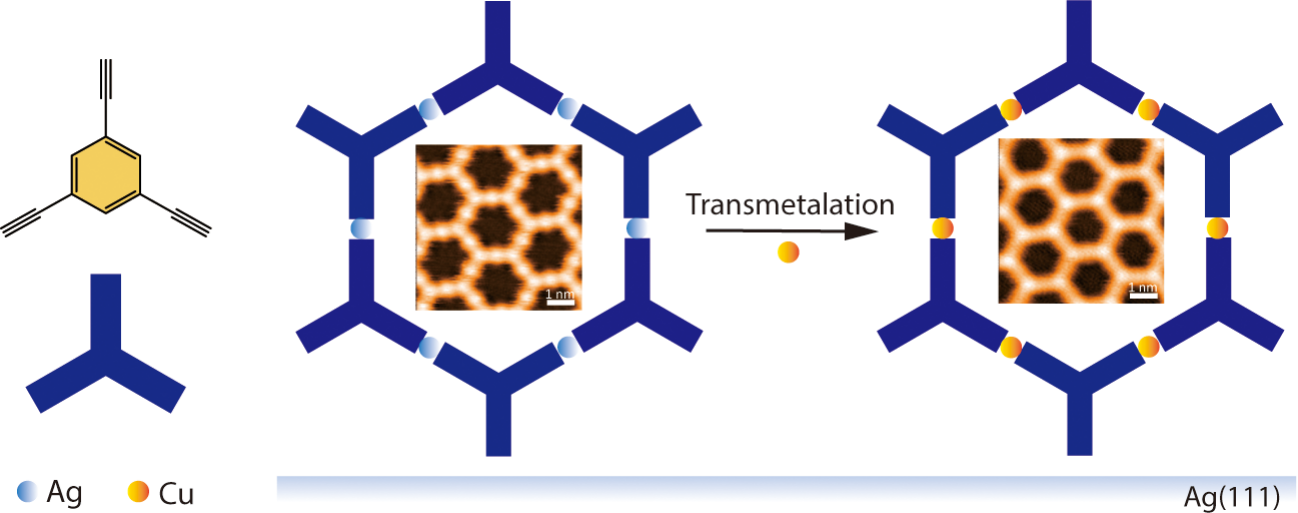

Transmetalation represents an appealing strategy toward fabricating and tuning functional metal–organic polymers and frameworks for diverse applications. In particular, building two-dimensional metal–organic and organometallic networks affords versatile nanoarchitectures of potential interest for nanodevices and quantum technology. The controlled replacement of embedded metal centers holds promise for exploring versatile material varieties by serial modification and different functionalization. Herein, we introduce a protocol for the modification of a single-layer carbon–metal-based organometallic network via transmetalation. By integrating external Cu atoms into the alkynyl–Ag organometallic network constructed with 1,3,5-triethynylbenzene precursors, we successfully realized in situ its highly regular alkynyl–Cu counterpart on the Ag(111) surface. While maintaining a similar lattice periodicity and pore morphology to the original alkynyl–Ag sheet, the Cu-based network exhibits increased thermal stability, guaranteeing improved robustness for practical implementation.

In recent years, the systematic development of two-dimensional (2D) molecule-based materials has become the focus of intense research owing to their unique physical and chemical properties.^1,2^ Supramolecular self-assembly and coordination engineering with organic molecules on surfaces represent powerful bottom-up strategies for the efficient construction of 2D molecular materials suitable for catalysis, magnetism, semiconducting electronic devices, and so forth,^1,3−7^ which can be for instance tailored to incorporate functional magnetic metal centers,^8^ such as Co,^9,10^ Ho,^11^ and Dy.^12^ A major challenge in this field is to increase system stability by designing 2D materials via covalent on-surface synthesis. Covalent linkages are advantageous because they can afford single-layer organic structures with delocalized electronic conduction and excellent robustness.^13−15^ The on-surface synthesis of 2D covalent nanoarchitectures is typically performed in a vacuum on atomically well-defined metal surfaces.^16−18^ However, this approach commonly faces the problem of limited extension of the ordered domains^19−21^ due to the occurrence of undesired reaction channels and the irreversibility of the covalent bond formation in a vacuum, which prevents the healing of defects.^22^

Organometallic bonding, mostly involving carbon–metal linkages, due to its generally covalent nature^23^ and the reversibility associated with the binding characteristics,^24^ may provide a powerful strategy for engineering surface-confined organic networks with appreciable robustness as well as impressive mesoscale regularity.^25,26^ 2D organometallic networks (OMNs) feature functional 2D materials with a significant promise. The often higher flexibility relative to purely covalent linking and the reversibility during the formation process in the low-dimensional environment of a surface offer the possibility of self-healing of defects, potentially affording higher order and tailored packing motifs and symmetry. Although various OMNs have been constructed with selected molecular building blocks via consuming surface adatoms provided by the substrate underneath, the success rate for their realization is often unforeseeable and strongly substrate-dependent, limiting the general applicability of such functional organometallic materials. In particular, it has recently been shown that extended graphdiyne-like, alkynyl–silver–alkynyl, and alkynyl–gold–alkynyl honeycomb OMNs can be prepared successfully by applying on-surface synthesis strategies.^25−29^ Yet, the large spectrum of metal centers and possible organic linkers calls for the identification of efficient routes to bestow the desired variety and versatility to the structural and electronic properties of such films.

Recently, there has been considerable interest in synthesizing, modifying, or functionalizing metal–organic frameworks (MOFs) by transmetalation via solution chemistry, with the aim to tailor their structures and performances for various applications.^30−32^ For instance, Li et al. utilized transmetalation method for the synthesis of an isomer MOF enhancing the catalytic performances.^33^ In-vacuum surface-adsorbed macrocycles, e.g., metalloporphyrins, have been reported to undergo transmetalation, facilitating the creation of macrocycles and the tuning of their properties for heterogeneous catalysis.^34−37^ Accordingly, in the context of surface-supported 2D OMNs, transmetalation appears as an appealing route for customizing the structure–property relationship of such attractive 2D materials in a controlled way. Surprisingly, this option has been rarely studied within the field of metallosupramolecular surface networks, with the exception of a nitrogen–metal linked 2D conjugated system.^38^ In particular, successful postsynthetic transformation of surface-confined 2D-OMNs with carbon–metal bonding, bearing significant prospects for appealing 2D graphyne- or graphdiyne-like materials with interesting electronic properties has not been reported so far.

Herein, combining a suite of surface science techniques, including scanning tunneling microscopy (STM), low-energy electron diffraction (LEED), and X-ray photoelectron spectroscopy (XPS), as well as density functional theory (DFT) calculations, we address the deliberate modification of single-layer OMNs from an alkynyl–Ag–alkynyl to an alkynyl–Cu–alkynyl linking platform via postsynthetic transmetalation. Using a simple 1,3,5-triethynylbenzene (TEB) precursor, extended organometallic honeycomb Ag-TEB networks are first synthesized on an Ag(111) surface under ultrahigh vacuum (UHV) conditions, by means of a previously reported gas-mediated surface reaction protocol.^25^ The subsequent deposition of Cu adatoms, followed by thermal annealing to appropriate temperatures, triggers the transformation of Ag-TEB to Cu-TEB OMN (Scheme 1). The latter 2D OMN, being not directly achievable on a Cu(111) substrate, possesses higher thermal stability, while retaining a similar lattice periodicity and pore symmetry as the original Ag-TEB. This finding provides a simple route to the engineering of a larger variety of single-layer robust carbon-based nanomaterials at well-defined conditions.

**Scheme 1:**
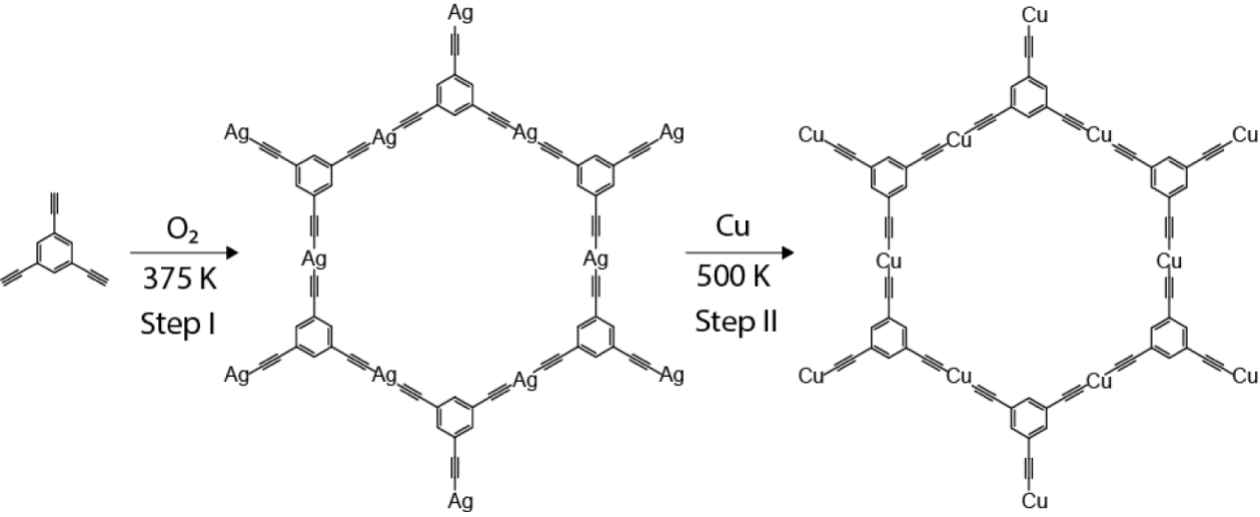
Schematic of the Ag-TEB OMN Preparation and Its Transformation into Cu-TEB OMN Step I: exposure to O_2_ at 300 K and postannealing at 375 K. Step II: Cu deposition at 300 K and postannealing at 500 K.

## Results and Discussion

### Modification of Ag-TEB OMN by Transmetalation Protocol to Fabricate Cu-TEB OMN

The self-assembly of the TEB precursor on Ag(111) was previously characterized by Kepčija et al.^20^ Here, we initially exposed a TEB submonolayer assembly to O_2_ (600 L; 1 *L* = 1.33 × 10^–6^ mbar·s) at 300 K, followed by annealing at 375 K. This preparation strategy (summarized in Scheme 1, step I) was successfully used in reference (25) to create a highly regular OMN with alkynyl–Ag–alkynyl linkages, using the more extended, 1,3,5-tris(4-ethynylphenyl)benzene (referred to as Ext-TEB) precursor.

**Figure 1:**
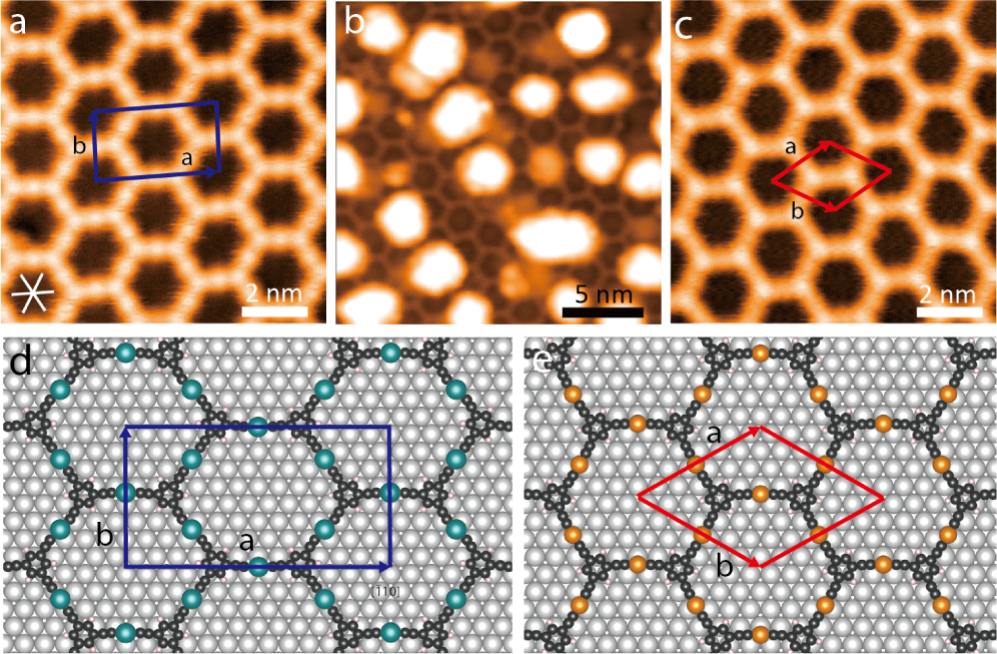
STM topography images of (a) Ag-TEB OMN formed after dosing O_2_ on TEB organic assembly on Ag(111) at 300 K and annealing at 375 K, (b) Cu clusters on Ag-TEB OMN before postannealing, and (c) Cu-TEB OMN obtained by dosing Cu atoms on Ag-TEB OMN and then postannealing to 500 K, inducing transmetalation. The surface unit cells are highlighted by blue and red frames, respectively. DFT simulated model of Ag-TEB OMN (d) and Cu-TEB OMN (e) on a silver slab (see the method). The images in (a) and (c) were taken with tunneling parameters of *V*_b_ = −100 mV, *I*_t_ = 1 nA, while the image in (b) was scanned at *V*_b_ = 380 mV, *I*_t_ = 300 pA. The closed-packed crystallographic directions of the substrate are denoted as white lines in panel (a).

As illustrated in Figure 1a, an ordered, porous Ag-TEB OMN readily evolves. Moreover, Figure S1a,b shows that the OMN grows into large domains extending up to micrometer size, similar to Ext-TEB on Ag(111).^25^ The blue frame in Figure 1a outlines the 2D centered unit cell of the Ag-TEB OMN, with lattice constant measured as *a* = 37.6 ± 0.2 Å, *b* = 20.7 ± 0.2 Å, and an angle of γ ≈ 90.2 ± 1°. More precisely, the Ag-TEB OMN structure displays two adjacent columns of neighboring, distorted hexagonal motifs, corresponding to a p2gg plane group with glide symmetry (Figure S1c), which is also similar to Ext-TEB.^25^ This renders the description of the periodicity through a quasi-rectangular unit cell more precise than using a smaller quasi-hexagonal rhombic primitive cell (indicated in Figure S1d, yellow rhombus) with primitive vectors (*a* = *b* ≈ 21.5 Å and γ ≈ 57.7°) joining the centers of adjacent (but distorted) hexagonal pores. Moreover, the 2D fast Fourier transform (2D-FFT), inset of Figure S1b, of a neat large-area patch also shows a rectangle-like unit cell. Panel b in Figure 1 characterizes the intermediate preparation step after depositing Cu on Ag TEB OMN at 300 K. The bright-appearing clusters are identified as agglomerates of Cu atoms adsorbed on top of the networks. After subsequently annealing this system at 500 K (Scheme 1, step II), a regular porous network with honeycomb symmetry and no superimposing clusters is restored, as illustrated in Figure 1c. The structure is similar to the original, pristine Ag-TEB OMN; however, it features differences in several details, whence we interpret the new network as Cu-TEB OMN formed by the establishment of alkynyl–Cu–alkynyl linkages (*vide infra*). The corresponding unit cell, depicted in Figure 1c, is marked by the red rhombus, whereby the lattice parameters are similar to each other in length, *a* = 20.1 ± 0.2 Å, *b* = 20.3 ± 0.2 Å, and the angle γ between unit cell vectors a and b amounts to 59.2 ± 1.0°. Consequently, the pores of Cu-TEB OMN come closer to the perfect hexagonal symmetry than Ag-TEB OMN, while exhibiting a slightly smaller pore size.

To elucidate the origin of the slight structural and morphological differences, herein, we performed DFT calculations. Specifically, gas phase DFT optimization for unit cell representation of the alkynyl–Ag–alkynyl and alkynyl–Cu–alkynyl linkages reveals that the length of Ag-TEB is larger than for the Cu-TEB linkage, as shown in Figure S2a,e. Moreover, Figure 1d,e depicts the DFT calculated structures for Ag-TEB and Cu-TEB OMN on Ag(111). The rectangular unit cell of Ag-TEB OMN in Figure 1d is outlined in blue, and the lattice constants of the Ag-TEB model are *a* ≈ 37.6 Å and *b* ≈ 20.0 Å, which are close to experimental unit cell constants *a* = 37.6 ± 0.2 Å, *b* = 20.7 ± 0.2 Å. The rhombic unit cell of Cu-TEB OMN in Figure 1e is marked in red. The unit cell constants of the Cu-TEB model are *a* = *b* ≈ 20.0 Å, which are close to experimental results (*a* = 20.1 ± 0.2 Å, *b* = 20.3 ± 0.2 Å). While Cu-TEB OMN can place all Cu atoms and the benzene rings at hollow sites of the Ag(111) surface well, Ag-TEB OMN needs to be stretched along the  direction due to the mismatch with the substrate. Besides, the binding energy for both OMNs was calculated (see Experimental Methods), the *E*_bind(Cu-TEB OMN)_ = −6.90 eV being lower than *E*_bind(Ag-TEB OMN)_ = −3.88 eV by about 3.0 eV, which in turn implies that the alkynyl–Cu–alkynyl linkage is significantly more stable than the alkynyl–Ag–alkynyl counterpart, and thus offers a possible driving force for the transmetalation from C–Ag–C to C–Cu–C connections (further discussed and rationalized in Figure S2).

Notably, in Figure 1c the midpoints on each side of the hexagonal outline, identified as embedded metal adatoms, display different contrasts with respect to the surroundings as compared to Figure 1a. Ag atoms appear distinctly brighter than Cu atoms at the same scanning bias. Figure S3a,b presents high-resolution STM images of the Ag-TEB and Cu-TEB OMN, respectively. As observed, with the same scanning parameters, on the edges of the honeycomb pore in the Ag-TEB OMN, three bright dots are visible. The dots located at the two ends of an edge (thus at the hexagon’s corners) are attributed to the benzene rings of each TEB molecule, whereas midpoint dots are identified as the Ag atoms. As for the Cu-TEB OMN, instead, the central dot of each single hexagon side is much dimmer than the adjacent benzene rings. These same appearances are revealed in close-up constant-height STM images of the single pore (Figure S3c,d). Accordingly, upon comparison of the lattice parameters as well as the general appearance of both OMNs before and after Cu addition, a smooth transformation from Ag-TEB to Cu-TEB OMN is inferred. As shown in Figure S4, the presence of large-area domain characteristic of Ag-TEB OMNs is retained upon transmetalation. Furthermore, excess Cu atoms gathered and grew into small Cu islands (Figure S4a) due to increased diffusion in the annealing process, promoting organization into a neat OMN at 500 K.

**Figure 2:**
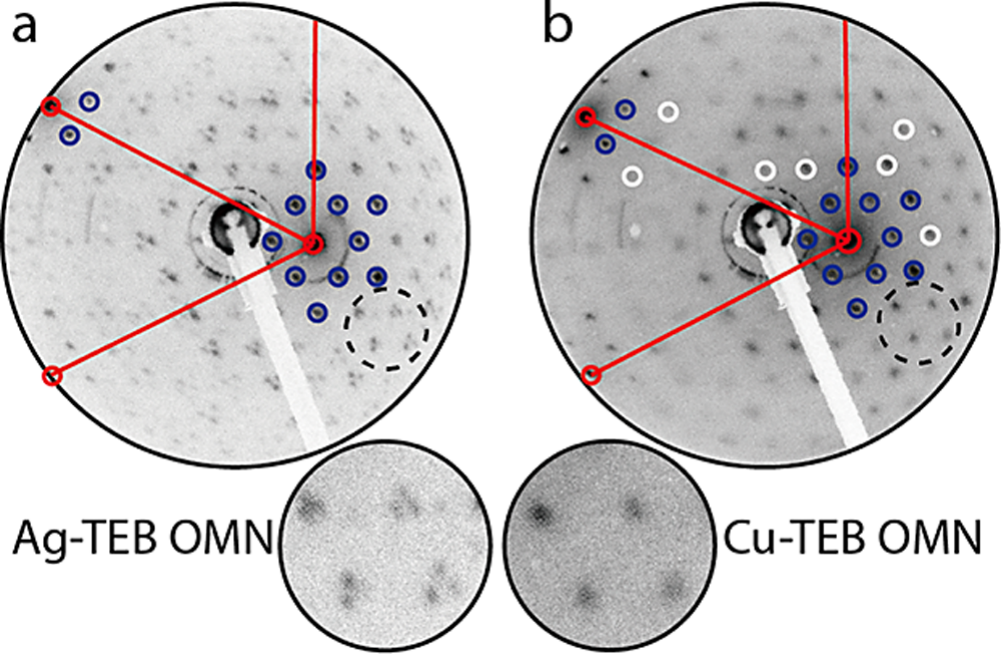
LEED patterns for Ag-TEB OMN (a) and Cu-TEB OMN formed after Cu deposition on deprotonated TEB and annealing at 500 K (b); the zero- and first-order diffraction spots are encircled in red and the  direction of real-space are marked by red lines. The insets show the magnification of partial pattern spots circled by black dashed lines. The LEED patterns were acquired at a primary electron energy of 30 eV.

Additional LEED measurements were performed to characterize the long-range order and superstructure periodicity of the surface preparations to further substantiate the transition from Ag-TEB to Cu-TEB OMNs. Figure 2a depicts the experimental LEED pattern acquired at 90 K for the pristine Ag-TEB OMN corresponding to the structure shown in Figure 1a. The LEED pattern in Figure 2b reflects the structure obtained after Cu deposition and annealing to 500 K. The spots highlighted by blue circles appear equally sharp for both OMNs, moreover, in the immediate vicinity of the zero-order spot, they outline a hexagon, in agreement with the quasi-hexagonal porous networks of Figure 1a,c. However, the spots marked by white circles in Figure 2b emphasize single spots that split into three distinct reflexes in the Ag-TEB OMN (Figure 2a), regardless of the primary electron energy (Figures S5,S6), which can also be observed clearly from the magnification of the partial patterns. These characteristic triplets from hexagonal symmetry denote multiple domains with slight distortion present in the Ag-TEB OMN, in agreement with the glide symmetry relation for adjacent distorted hexagonal columns, which is absent for the Cu-TEB OMN. The subtle change in lattice periodicity is thus reflected in a clear difference in LEED characteristics for the two distinct OMNs, as evidenced in Figure 2 and further summarized in Figures S7,S8. Besides, the LEED patterns of the two OMNs after annealing at the same temperature of 450 K are reported in Figure S9.

**Figure 3:**
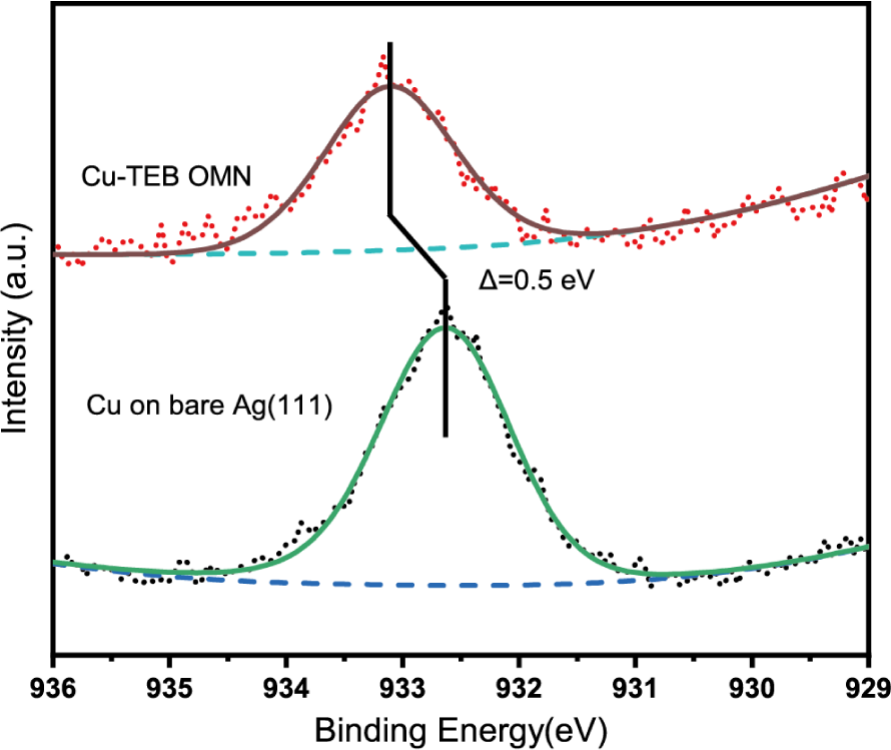
XPS measurements of the Cu 2p_3/2_ core level for the Cu-TEB OMN after annealing at 500 K (top) and for Cu deposited onto pristine Ag(111) at 300 K (bottom). The experimental data are shown as dots, the black and green lines denote fitted curves, and the dashed blue lines represent the secondary electron background. The chemical shift of 0.5 eV upon Cu incorporation into the OMN is highlighted by vertical lines.

Chemical insight supporting the transmetalation was obtained by XPS. Figure 3 depicts the Cu 2p_3/2_ core-level spectrum of the Cu-TEB OMN (top), recorded after annealing at 500 K, which is compared to the spectrum of metallic copper upon Cu deposition onto pristine Ag(111) (bottom). These spectra reveal that the Cu 2p_3/2_ peak position undergoes a binding energy shift of 0.5 eV, which is attributed to the organometallic linkages expressed. In addition, a weak component attributed to residual metallic Cu (unreacted) is found after peak fitting (Figure S10a). For reference, note that the Cu 2p_3/2_ binding energy position does not change for Cu deposited on pristine Ag(111) after annealing at 450 and 500 K (Figure S10b). This advocates the conclusion that the chemical state of deposited Cu atoms has changed due to the interaction with TEB molecules, leading to alkynyl–Cu–alkynyl linkages, and not simply due to the thermal evolution of the Cu atomic environment on Ag(111).

Furthermore, we also assessed for comparison an alternative method to form the Cu-TEB OMN without the initial preparation of the Ag-TEB OMN (Figure S11). Cu atoms were deposited on deprotonated TEB directly, followed by thermal annealing the system at 450 K. Strikingly, a smaller yield of Cu-TEB OMN occurred compared to Figure 1c, which signals that the transmetalation approach (using the protocol shown in Scheme 1) is more effective and flexible.

### Confinement of Electronic Surface States in Ag-TEB and Cu-TEB OMNs

**Figure 4:**
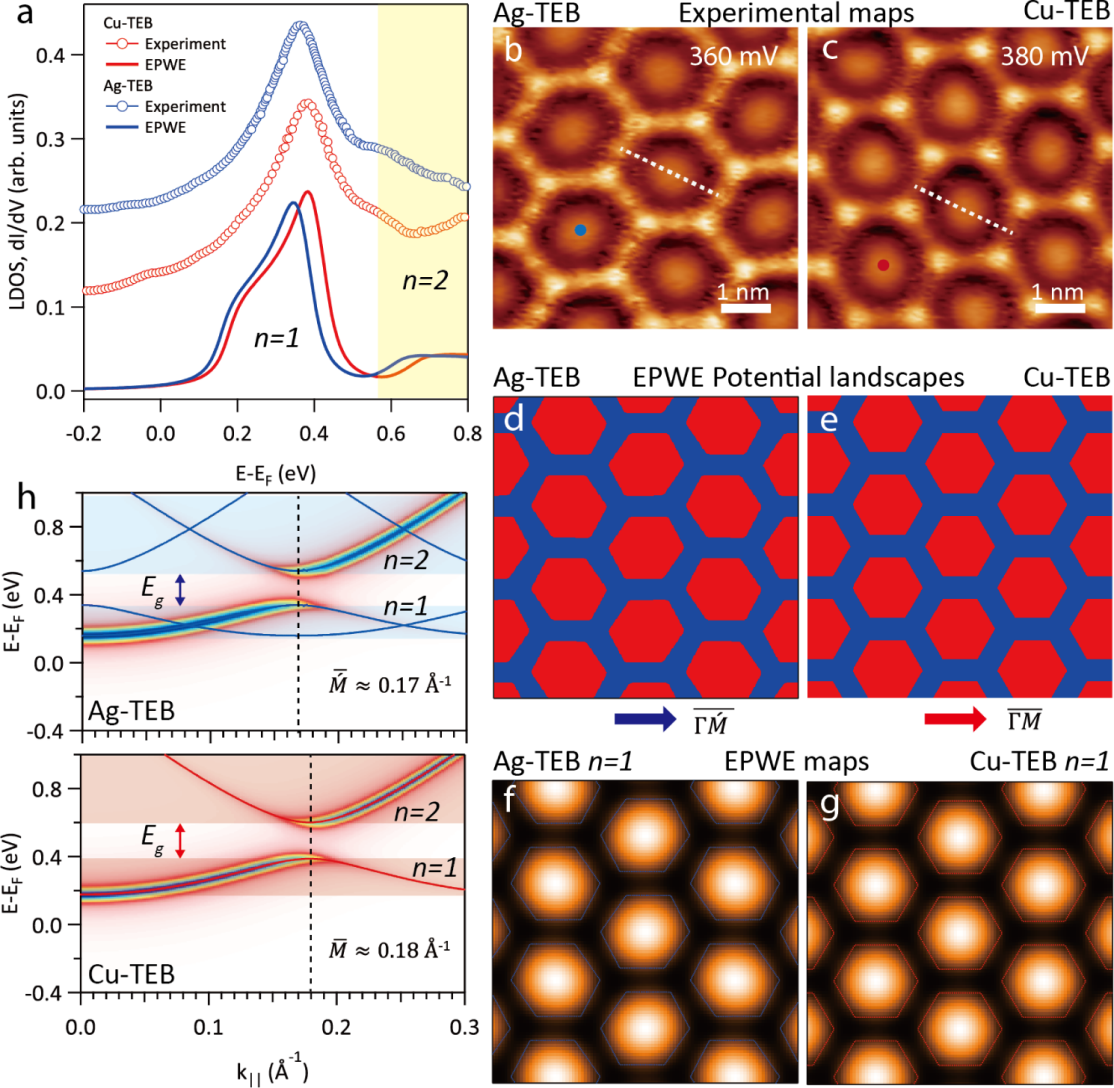
Surface state confinement in Ag-TEB and Cu-TEB OMNs. Panel (a) compares the calculated LDOS from EPWE (electron plane wave expansion) simulations with the experimental d*I*/d*V* spectra. Panels (b) and (c) present the d*I*/d*V* mapping of Ag-TEB and Cu-TEB OMN at 360 and 380 mV, respectively. The tip positions for STS spectra are marked by blue and red dots in parts (b) and (c). EPWE simulation potential geometry for Ag-TEB (d) and Cu-TEB OMN (e). LDOS maps of the (f) Ag-TEB OMN at 360 mV and (g) Cu-TEB OMN at 380 mV. (h) EPWE surface state band structure (blue and red) and photoemission spectral weight of Ag-TEB and Cu-TEB OMN. Panel (b) was scanned at *V*_b_ = 360 mV, and (c) at *V*_b_ = 380 mV.

Adsorbed nanoporous networks are known to mediate quantum confinement phenomena, notably with surface state electrons at fcc(111) coinage metal surfaces. Since these electrons are sensitive to the pore size and shape, we studied their confinement to get further insights into the electronic characteristics of the two systems. Scanning tunneling spectra (STS) were measured at the pore centers of both networks with biases between −200 and 800 mV. These results are depicted in Figure 4a, which presents the respective experimental d*I*/d*V* conductance curves for Ag-TEB (blue data) and Cu-TEB (red data). For the Ag-TEB OMN one discerns a peak centered at about 362 mV reflecting a resonance at the pore center, while for the Cu-TEB OMN, the maximum is distinctly blueshifted to 386 mV, i.e., by more than 20 mV (see Figure S12 for long-range STS spectra from −1.0 to 1.5 V). Moreover, constant-height d*I*/d*V* maps at 360 mV and 380 mV offer distinctly identifiable confinement states in the pores of Ag-TEB and Cu-TEB OMNs, respectively, as shown in Figure 4b,c (the original, constant-height STM topographic images are shown for reference in Figure S13a,b).^39−41^

To better understand the origin of the electronic differences, we focus on the slightly different pore sizes upon transmetalation. Accordingly, semiempirical electron plane wave expansion (EPWE) simulations of both OMNs are utilized to unravel the origin of the observed blue shift. The potential landscapes for EPWE modeling were constructed according to the topographic images and are shown in Figure 4d,e. The red regions stand for the Ag(111) substrate with zero potential, and the blue areas correspond to potential barriers (V) set at the position of the metal–organic backbone in Ag-TEB (Figure 4d) and Cu-TEB OMN (Figure 4e). The barrier width at the sides of the honeycomb OMN is fixed to 5.0 Å, which is estimated from experimental high-resolution STM images, and the OMN structures are considered to exert a repulsive potential to surface electrons. We define the surface state reference *E*_ref_ = −0.058 eV, *V* = 0.6 eV, and the effective mass *m*_eff_ = 0.39 *m*_e_, as the calculation parameters.^42^ For the sake of simplicity, the simulation method neglects the contribution originating from the two different kinds of metal atoms, keeping the focus on the size and deformation of the two pores. As a result, the calculated local density of states (LDOS) spectral features agree well with the experimental d*I*/d*V* curves, where the experimental shift is well reproduced by the EPWE simulations, as proven in the bottom part of Figure 4a. Meanwhile, Figure 4f,g provides the calculated LDOS maps with corresponding energy at 360 mV for Ag-TEB OMN, and 380 mV for Cu-TEB OMN, which are in good agreement with Figure 4b,c, respectively.

In addition, according to the calculation by EPWE, we obtained the surface state band structures of Ag-TEB OMN and Cu-TEB OMN as indicated in Figure 4h. The band structures consist of two main bands separated by an energy gap for the two types of OMNs. The additional bands for Ag-TEB originate from the conventional centered rectangular unit cell enclosing two pores, but they carry no spectral weight as demonstrated by the simulated photoemission intensity.^43−45^ Therefore, consistent with LEED observations, the photoemission spectral weight follows a quasi-hexagonal lattice with slightly different M′ and M points for Ag-TEB and Cu-TEB. The low energy bands (*n* = 1) correspond to a bound state with some coupling corresponding to 360 and 380 mV, while the bands above the energy gap (*n* = 2), represent the scattering state above the barrier energy corresponding to 660 and 680 mV respectively. The EPWE simulated LDOS map at 660 eV for Ag-TEB OMN and 680 eV for Cu-TEB OMN are shown in Figure S13c,d respectively, revealing weak donut-like shapes typical for *n* = 2 confined states.

In order to gain more detailed electronic information on the pores, line STS were acquired across the pores along a line through the pore center crossing two metal atoms (white dashed lines drawn in Figure 4b,c). From the STS color-coded contour plots, as shown in Figure S13e,g the strong central maxima located at 362 and 386 mV are observed, whereby EPWE simulations for Ag-TEB and Cu-TEB OMN (Figure S13f,h) agree well with the experimental results. Therefore, the STS peak shift of the confined state further supports the formation of a Cu-TEB OMN from the original Ag-TEB OMN.

The bias-dependent STM images of both OMNs are also compared in Figures S14 and S15. TEB benzene ring parts located at the vertex of both OMNs and Ag atoms at the midpoint of pores sides are imaged as bright protrusions, with brightness varying with the bias voltage changes. However, Cu metal atoms were imaged to be darker than benzene rings. In both OMNs, the *n* = 1 confined surface state inside the pores can be apparently imaged at 0.5 V which is consistent with the d*I*/d*V* curves.

### Thermal Stability and Induction of Alkynyl–Alkynyl Homocoupling

Besides the electronic differences, the Cu-TEB OMN exhibits a thermal stability distinctly higher than that of the Ag-TEB counterpart. Figure S16a shows that the Ag-TEB OMN decomposes after annealing at or below 500 K. Conversely, the Cu-TEB OMN is preserved intact, even after annealing at 600 K, as demonstrated in Figure S16b. This enhanced stability further corroborates the successful transformation from the Ag-TEB OMN to the distinctly more robust Cu-TEB OMN. Based on the higher stability of the Cu-TEB network, the possible covalent homocoupling of alkynyl groups can be explored by further thermal treatment. This may introduce alternative suggestions to the engineering of single-layer graphdiyne materials under controlled conditions, since the debate of (non)dehydrogenative alkyne coupling scenario on noble metal surfaces signals potential difficulties when targeting alkynyl–alkynyl connections obtained from terminal alkynes.^46,47^

**Figure 5:**
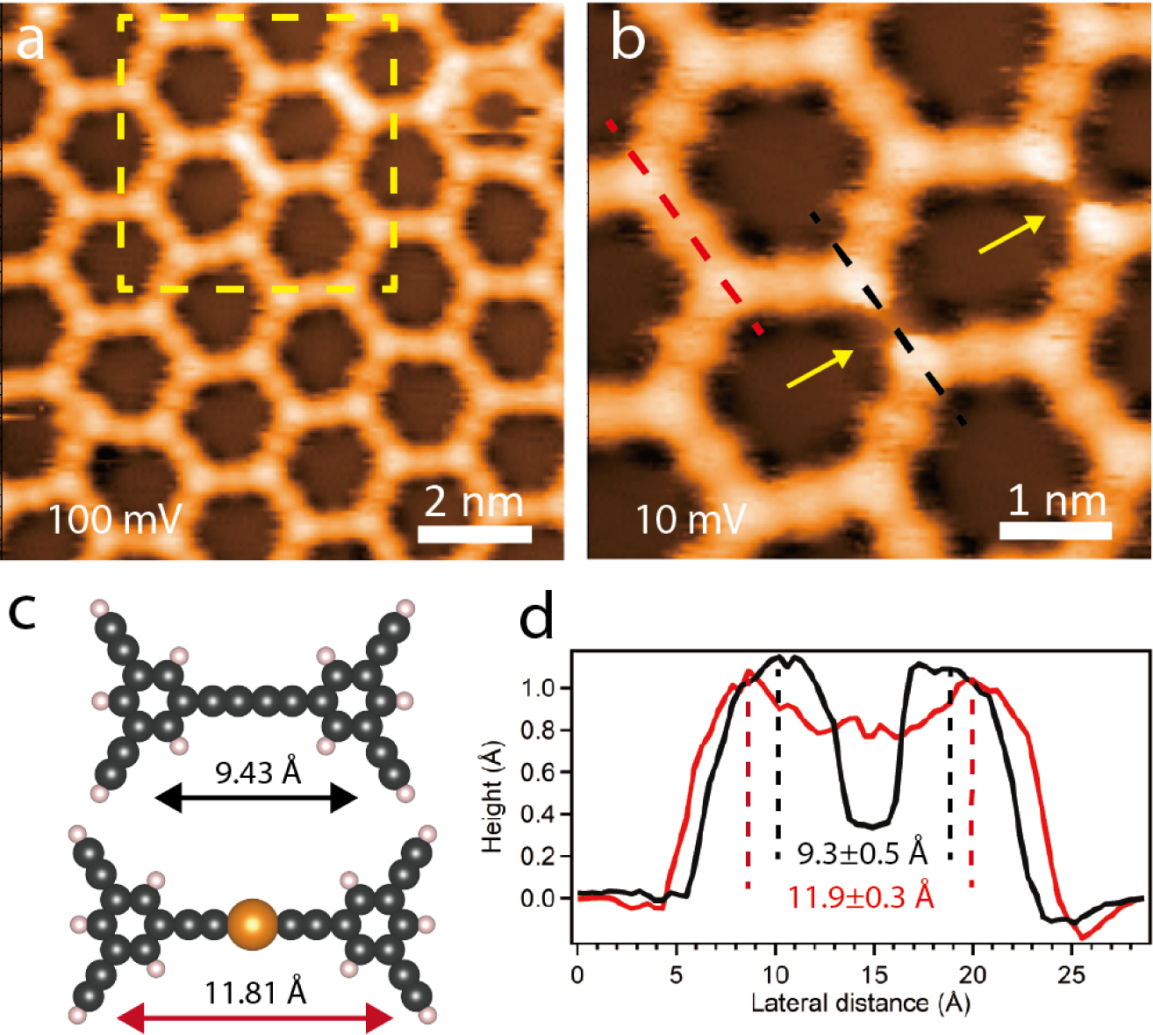
(a) Large-area and (b) zoom-in STM images of a Cu-TEB OMN with alkynyl–alkynyl coupling structures, scanned at *V*_b_ = 100 mV, *I*_t_ = 100 pA (a) and *V*_b_ = 10 mV, *I*_*t*_ = 100 pA (b), respectively. (c) DFT Optimized model of alkynyl–alkynyl (top) and alkynyl–Cu–alkynyl (bottom) coupling of TEB dimers in the gas phase. (d) Experimental profiles measured along alkynyl–alkynyl (black) and alkynyl–Cu–alkynyl coupling (red) direction.

Indeed, upon annealing the Cu-TEB network up to 500 K, the STM image in Figure 5a shows that some edges of hexagonal pores in the Cu-TEB OMN (inside the dashed yellow region) are occasionally imaged brighter than others. The zoomed-in STM image of Figure 5b acquired coincidentally with a special tip reveals the missing central bright dot at lower bias (indicated by arrows), at variance with the typical appearance of the alkynyl–Cu–alkynyl bridges. This structure is associated with alkynyl–alkynyl homocoupling schematically depicted in Figure 5c, where the DFT optimized model of the alkynyl–Cu–alkynyl linkage and the alkynyl–alkynyl coupling structure in the gas phase are compared. As the length of the former is 11.81 Å and that of the latter 9.43 Å, the alkynyl–alkynyl distance is shortened by 2.38 Å. As indicated in Figure 5d, also in the STM data, the length of the two structures across their line profile (dashed red and black lines) differs. Referring to the maxima, the red (11.9 ± 0.3 Å) is about 2.6 Å longer than the black connection (9.3 ± 0.5 Å), signaling a modified bonding motif, tentatively assigned to alkynyl homocoupling.

The increased thermal stability of the Cu-TEB OMNs facilitates the initiation of alkynyl–alkynyl homocoupling at elevated conditions. However, either extending the annealing time to 1 h at 500 K or increasing the annealing temperature to 600 K (Figure S17a,b) does not yet promote the large structure of diacetylene linkages. Moreover, the network started decomposing with a long annealing time or higher annealing temperature. One promising strategy might be the introduction of a gas atmosphere as a reducing agent during thermal treatment. Figure S17c reports a larger-area image where more bright segments attributed to alkynyl–alkynyl coupling appeared after annealing at 500 K in an ammonia atmosphere. The latter was used deliberately as a reducing agent. In Figure S17d, even hexagons with two modified linkages could be observed, i.e., without the typical protrusions encountered for alkynyl–Cu–alkynyl linkages. Bias-dependent images for the structure of Figure S17d are shown in Figure S18, the alkynyl coupling part being resolved as a bright rod, while other hexagonal sides were “normal” when imaged at voltages below 1.0 V. In contrast, the alkynyl coupling segment splits into two protrusions that are located at the benzene positions, and coupling bond sites turn dark for bias above 2.0 V. Thus, the successful occurrence of alkynyl–alkynyl homocoupling is likely, in marked contrast to the case of the Ag-TEB OMN for which disruption of the network prevails at elevated temperatures over the covalent homocoupling (Figure S16a). Thus, the OMN transmetalation bears promise as a viable approach toward obtaining in a multistep reaction scheme regular all-carbon graphdiyne networks on surfaces. Nevertheless, there may be deeper factors deserving further systematic investigation in order to explore a rationale regarding transmetalation protocols for surface-confined OMNs, such as the accessible oxidation states of the metal atoms, the diverse ability of multifold coordination in the preexisting structural motif, as well as different carbon–metal interaction strengths (Figure S19).

## Conclusions

We have explored the modification of a surface-confined single-layer OMN by transmetalation, specifically generating from an alkynyl–Ag–alkynyl to an alkynyl–Cu–alkynyl linking platform via postsynthetic reaction. Specifically, the deposition of Cu adatoms on extended organometallic honeycomb Ag-TEB networks followed by thermal annealing to appropriate temperature induces the distinct transformation of Ag-TEB into Cu-TEB OMN. The latter structure is proven to possess higher thermal stability while maintaining a similar lattice periodicity and pore symmetry as the Ag-TEB OMN. Our work demonstrates an effective strategy to systematically prepare OMNs incorporating selected metal species and providing increased thermal stability, maintaining higher robustness for prospective applications.

## Experimental Methods

### Materials

1,3,5-Triethynylbenzene (TEB) was purchased from Sigma-Aldrich with a purity of 97%. Cu atoms were deposited with amounts of 1–5% ML by a homemade evaporator with metal wire. The copper wire was supplied by GoodFellow GmbH and was wound around a central tungsten wire. The power output voltage was adjusted via an amplifier to make the wire glowing and obtain a steady flux, such that the Cu deposition could be controllable and reproducible.

### Sample Preparation

Sample preparations were performed in a UHV setup with a base pressure below 2.0 × 10^–10^ mbar. All STM measurements were performed using a commercial Joule-Thomson STM. The Ag(111) surface was cleaned by Ar^+^-sputtering cycles at 0.9 kV, followed by annealing at 730 K. The tungsten tip was prepared through electrochemical and sputtering-annealing cycles. All measurements were performed at 4.5 K. STM images were analyzed by Igor software. Figure 4b,c, among them, is processed by WSxM software.^48^ The precursor TEB powder was loaded into a glass vial connected to a needle via a flange with an attached leak valve in order to finely control the molecule flow. After evaporating the appropriate amount of molecules onto the surface at room temperature, the sample was exposed to O_2_ atmosphere (∼1.33 × 10^–6^ mbar, 60 s) and subsequently annealed at 375 K. Afterward, Cu was deposited on the surface at room temperature, followed by annealing at 500 K to obtain an ordered and clean Cu-TEB OMN.

### X-ray Photoelectron Spectroscopy (XPS) and Low-Energy Electron Diffraction (LEED)

All XPS and LEED experiments were conducted in a home-built UHV chamber. The XPS measurements were performed using the nonmonochromatized Mg Kα radiation line (*hν* = 1253.6 eV) and a SPECS Phoibos 100 CCD hemispherical analyzer. Experimental LEED patterns were acquired at 90 K using an OCI Vacuum Microengineering (BDL800IRLMX-ISH) apparatus. Casa XPS software (Casa Software Ltd., Teignmouth, UK) was used for the XPS data analysis and peak fitting.

### Electron Plane Wave Expansion (EPWE) Calculations

The EPWE method was utilized to model the local density of states (LDOS) and band structures for Ag-TEB and Cu-TEB OMNs. The potential landscapes are constructed as distinct regions of muffin-tin potentials defining the OMNs (V) and the pristine substrate, as shown in Figure 4. The Fourier coefficients of these predefined potentials (*V*_g_) are fed into the EPWE Hamiltonian matrices and are then solved for the eigenvalues and eigenvectors.^40,42,49^ We used a basis set consisting of ∼50 plane waves by terminating the potential expansion at a maximum number of reciprocal lattice vectors *G*_max_ = 7. A Gaussian broadening of 30 meV is applied to all electronic states. The EPWE code used in this study is developed by Prof. Javier GarcÍa de Abajo.

### Modeling Optimization

Alkynyl homocoupling and alkynyl–Cu–alkynyl structures were built and the geometry was optimized using DFT gas-phase calculation with the ORCA quantum chemistry code,^50^ at the B3LYP/def2-TZVP level with in-plane constraint.

### Periodic Structures with DFT

We employed DFT^51^ within the Kohn–Sham formalism using the QuickStep module^52^ built in the package CP2K (https://www.CP2K.org/). We utilized the generalized gradient approximation of Perdew, Burke, and Ernzerhof^53^ as the approximation to the exchange-correlation term and the empirical potential D3^54^ to account for the missing London dispersion forces, PBE+D3. Our calculated lattice constant for Ag is 4.084 Å. We modeled the Ag(111) surface with a slab of five layers of the substrate, with the two topmost layers allowed to relax, and H passivation on the bottom surface. We used an equidistance mesh of 4 × 4 *k* points in the hexagonal cell and of 2 × 4 *k* points in the rectangular cells in the integrals over the first Brillouin zone. Pseudo potentials,^55^ with a valence of 11 electrons on Cu and Ag, were employed together with Gaussian basis sets DZVP-MOLOPT^56^ to expand the wave functions and plane wave sets up to a cutoff energy of 700 Ry to expand the electron density. *E*_binding_ = *E*(network, sub) – 2*E*(isolated, sub) −3*E*(metal, sub), where *E*(network, sub) is the total energy of the network on the substrate (sub), *E*(isolated, sub) is the total energy of the isolated molecule on the substrate, and *E*(metal, sub) is the total energy of the adatom on the substrate. To compare their binding energies, we use a rhombic unit cell comprising two molecules and three metal adatoms for both networks.

## Supporting Information Available

Additional STM images and LEED patterns analysis; further discussion of DFT simulated models for Ag-TEB and Cu-TEB OMN; comparison of XP spectra for Cu on Ag(111) at different temperatures; discussion for an additional method of Cu-TEB OMN preparation; additional EPWE calculation results; additional STM images of alkynyl–alkynyl coupling linkage (PDF)
