# Supplementary material for: Transmetalation in Surface-Confined Single-Layer Organometallic Networks with Alkynyl–Metal–Alkynyl Linkages
Source: ACS Nano. 2024 Jul 23;18(31):20157–66. doi: 10.1021/acsnano.4c02263 (PMC11308921; doi:10.1021/acsnano.4c02263)
Supplement: Supplementary file 1 — nn4c02263_si_001.pdf [file nn4c02263_si_001.pdf]

Supporting Information for:

Transmetalation in Surface-Confined Single-Layer Organometallic Networks with Alkynyl-Metal-Alkynyl Linkages

Wenchao Zhao,[†,#] Felix Haag,[†,#] Ignacio Piquero-Zulaica,*[,†] Zakaria M. Abd El-Fattah,[‡,§]

Prashanth Pendem,[†] Pablo Vezzoni Vicente,[†] Yi-Qi Zhang,[†,⫽] Nan Cao,[†] Ari Paavo Seitsonen,[⊥]

Francesco Allegretti,*[,†] Biao Yang,*[,†,¶] and Johannes V. Barth[†]

[†] Physics Department E20, TUM School of Natural Sciences, Technical University of Munich, James Franck Straße 1, 85748 Garching, Germany

[‡] Physics Department, Faculty of Science, Al-Azhar University, Nasr City, 11884 Cairo, Egypt

[§] Physics Department, Faculty of Science, Galala University, New Galala City, Suez, 43511, Egypt

[⫽] Institute of Physics, Chinese Academy of Sciences, 100190, Beijing, China

[⊥] Département de Chemie, École Normale Supérieure, 24 rue Lhomond, Paris, F-75005 France

[¶] Institute of Functional Nano and Soft Materials (FUNSOM), Jiangsu Key Laboratory for Carbon Based Functional Materials and Devices, Soochow University, 199 Ren'ai Road, Suzhou 215123, Jiangsu, China

* Corresponding authors

Figure Contents

Figure S1. Large-area STM images of Ag-TEB OMN.

Figure S2. DFT Calculated models of Ag-TEB and Cu-TEB.

Figure S3. Detailed STM images and single pores of Ag-TEB and Cu-TEB OMN.

Figure S4. Large-area STM images of Cu-TEB OMN.

Figure S5. LEED patterns of Ag-TEB OMN acquired at 90 K with different primary electron energy.

Figure S6. LEED patterns of Cu-TEB OMN acquired at 90 K with different primary electron energy.

Figure S7. Simulated LEED patterns for Ag-TEB and Cu-TEB OMN and corresponding STM images.

Figure S8. Original LEED pattern of Ag-TEB OMN and Cu-TEB OMN with primary electron energy of 30 eV.

Figure S9. LEED patterns for Ag-TEB and Cu-TEB OMN after annealing at 450 K.

Figure S10. Peak fit of Cu $2p_{3/2}$ XP spectrum for Cu-TEB OMN and Cu $2p_{3/2}$ XPS measurements of Cu on pristine Ag(111) annealed to different temperatures.

Figure S11. STM images of deprotonated TEB self-assembly and Cu addition on deprotonated TEB self-assembly at 200 K, then annealing at 450 K.

Figure S12. Long-range d*I*/d*V* spectra from -1.0 V to 1.5 V.

Figure S13. Constant height STM images, theory LDOS maps and STS line spectra for Ag-TEB and Cu-TEB OMN.

Figure S14. Bias-dependent STM images for Cu-TEB OMN.

Figure S15. Bias-dependent STM images for Ag-TEB OMN.

Figure S16. STM images Ag-TEB OMN and Cu-TEB OMN after annealing.

Figure S17. STM images of alkynyl coupling structure.

Figure S18. Bias-dependent STM images of alkynyl coupling structure.

Figure S19. LEED patterns for Bi deposition onto Ag-TEB OMN and Ni onto Ext-TEB OMN.

Other used materials.

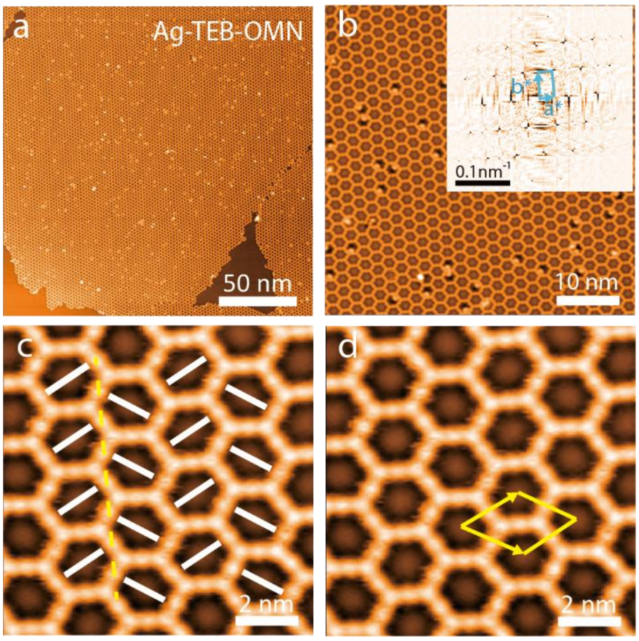

Figure S1. *Large-area STM images of Ag-TEB OMN with 0.2 µm-size (a) and 50 nm-size (b); the inset presents the 2D-FFT of the 50 nm × 50 nm area shown in panel (b). Panel (c) emphasizes the glide symmetry of neighboring distorted hexagons. In panel (d) we outline a rhombic unit cell (in yellow) similar to the Cu-TEB network of Figure 1c, as a comparison to the rectangle unit cell assumed in Figure 1a. The tunneling parameters are V_b = -500 mV, I_t = 100 pA (a); V_b = 400 mV, I_t = 1 nA (b); and V_b = 350 mV, I_t = 300 pA (c) and (d).*

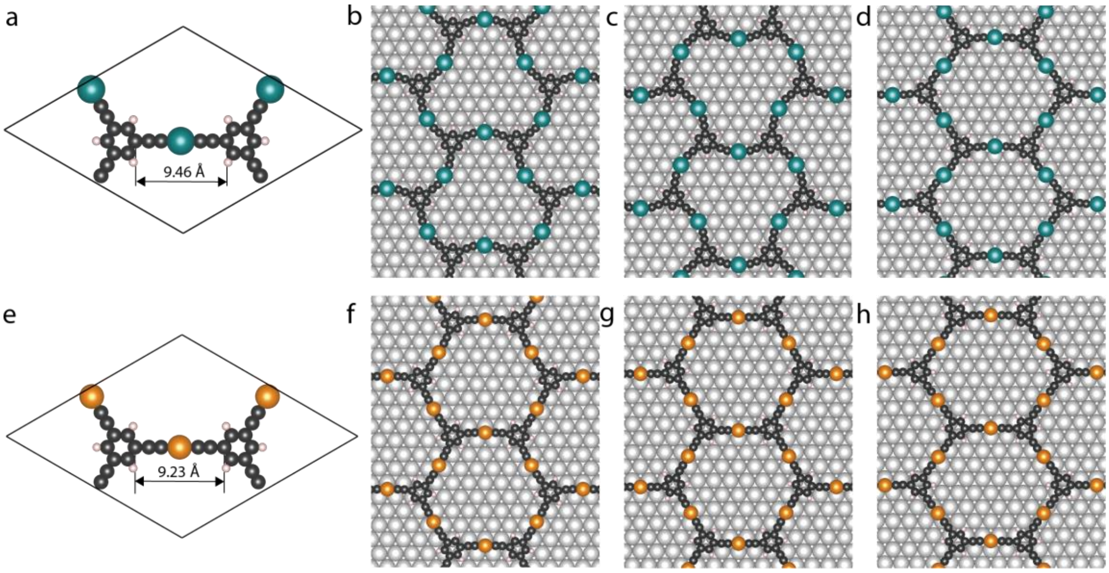

Figure S2. *Gas phase optimized structure for unit cell representation of Ag-TEB (a). DFT simulated models of Ag-TEB OMN with possible hexagonal, rectangular-12 and rectangular-13 unit cells (b) (c) (d) on the Ag(111) surface. Gas phase optimized structure for unit cell representation of Cu-TEB (e), and DFT simulated models of Cu-TEB OMN with possible hexagonal, rectangular-12 and rectangular-13 unit cells (f) (g) (h) on the Ag(111) surface. In*

the OMNs, the silver, copper, carbon and hydrogen atoms are marked by green, orange, black and pink spheres, respectively.

Firstly, the gas phase DFT optimization for unit cell representation of the alkynyl-Ag-alkynyl and alkynyl-Cu-alkynyl linkage reveals that the length of Ag-TEB is longer than the Cu-TEB linkage, as shown in Figure S2a and S2e. Secondly, we proposed and built calculated models as shown in Figure S2, with possible unit cells: hexagonal unit cells with vectors $4 \times \sqrt{3}$ in Figure S2b and S2f (hexagonal), rectangular unit cells with vectors $12 \times \sqrt{3}$ in Figure S2c and S2g (rectangular-12) and rectangular unit cells with vectors $13 \times \sqrt{3}$ in Figure S2d and S2h (rectangular-13), to simulate both networks adsorbed on the Ag(111) substrate by DFT. The rectangular-12 is in principle the same as the hexagonal one with two unit cells of the hexagonal cell inserted into the rectangular cell. The rectangular-13 has one more row of Ag substrate to be extended. Additionally, the formation energy for all network modes was calculated per two molecules and three metal atoms. The Ag atoms are coded in green and the Cu atoms are marked in orange.

In the case of Ag-TEB OMN, Figure S2b-d reveal the calculated hexagonal, rectangular-12 and rectangular-13 models for Ag-TEB OMN with $Ebind_{(Ag/hexagonal)}$ = -3.66 eV, $E_{bind(Ag/rectangular-12)}$ = -3.69 eV, and $E_{bind(Ag/rectangular-13)}$ = -3.88 eV, which implies rectangular-13 model the lower energy for Ag-TEB OMN and therein the most stable structure as shown in Figure S2d. Additionally, as shown in the hexagonal (Figure S2b) and the rectangle-12 (Figure S2c) structure, the Ag-TEB is fully distorted and doesn`t match our experimental topology images. Moreover, the lattice constants for Ag-TEB rectangular-13 model are a $\approx$ 37.6 Å and b $\approx$ 20.0 Å, which are also reasonably close to the average experimental results (a = 37.6 $\pm$ 0.2 Å; b = 20.7 $\pm$ 0.2 Å).

In the case of the Cu-TEB OMN model, the hexagonal, rectangular-12 and rectangular-13 models are presented in Figure S2f-h, respectively. The corresponding $E_{bind(Cu/hexagonal)}$ = -6.90 eV, $E_{bind(Cu/rectangular-12)}$ = -6.91 eV and $E_{bind(Cu/rectangular-13)}$ = -6.68 eV are found, which implies Figure S2f to be the most stable structure for Cu-TEB OMN. As we discussed before, hexagonal and rectangle-12 model are in principle similar, hence the formation energy for both are close, while also implies no distortions and nice matching between Cu-TEB OMN and Ag(111) substrate. The lattice constants for hexagonal one of Cu-TEB (Figure S2f-g) are a = b $\approx$ 20.0 Å, which are close to the average experimental results (a = 20.1 $\pm$ 0.2 Å, b = 20.3 $\pm$ 0.2 Å).

Comparing the rectangular-13 structure of Ag-TEB with the hexagonal one and rectangular-12 ones of Cu-TEB OMN, all the Cu atoms can be located at the hollow position (we show the case of hcp hollow sites for hexagonal one in Figure S2f, but fcc hollow sites for rectangular-12 in Figure S2g). Moreover, the benzene ring centers also seat at hollow positions. This reveals an excellent matching between the Cu-TEB OMN and the Ag(111) substrate. However, in the case of the Ag-TEB OMN (cf. Figure S2g) one cannot place all metal atoms at the same substrate sites in an extended ordered hexagonal network. The simulated model thus gives proof of a mismatch between the Ag-TEB OMN and the Ag(111) substrate, which causes some distortion in the network, and leads to the rectangular unit cell rather than the rhombic counterpart of the hexagonal structure. Besides, the alkynyl-Ag-alkynyl structure is flexible as described in previous study[1-3]. Therefore, the Ag-TEB OMN may be distorted due to this mismatch, whereas the Cu-TEB tends remain hexagonal.

In the following, we proceed to explain the principle driving force inside of the transmetalation on the surface focusing formation energy for the alkynyl-M-alkynyl structures. The DFT calculated formation energy for Ag-TEB and Cu-TEB OMN was apparently different. $E_{bind(Ag/rectangular-13)}$ = -3.88 eV for Ag-TEB OMN, and $E_{bind(Cu/hexagonal)}$ = -6.90 eV for Cu-TEB OMN. Thus, one can clearly see that the formation energy for alkynyl-Cu-alkynyl is lower than for alkynyl-Ag-alkynyl by 3.0 eV, which emphasizes that alkynyl-Cu-alkynyl is significantly more stable than alkynyl-Ag-alkynyl linkage.

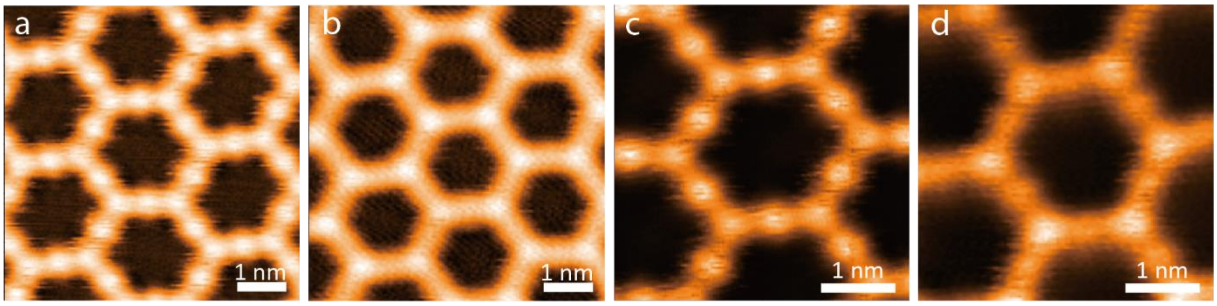

Figure S3. *High-resolution STM images of (a) Ag-TEB and (b) Cu-TEB OMN with constant current scanning mode. Scanning parameter for (a) and (b): V_b = -10 mV, I_t = 1 nA. Zoom-in STM images of a single pore of Ag-TEB (c) and Cu-TEB OMN (d) recorded at constant height scanning mode at V_b = 10 mV.*

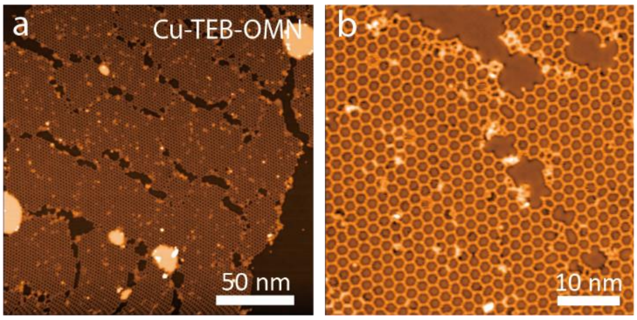

Figure S4. *Large-area STM images of Cu-TEB OMN with 0.2 µm-size (a) and 50 nm-size (b). The tunneling parameters are V_b = -1 V, I_t = 300 pA (a); V_b = 380 mV, I_t = 100 pA (b).*

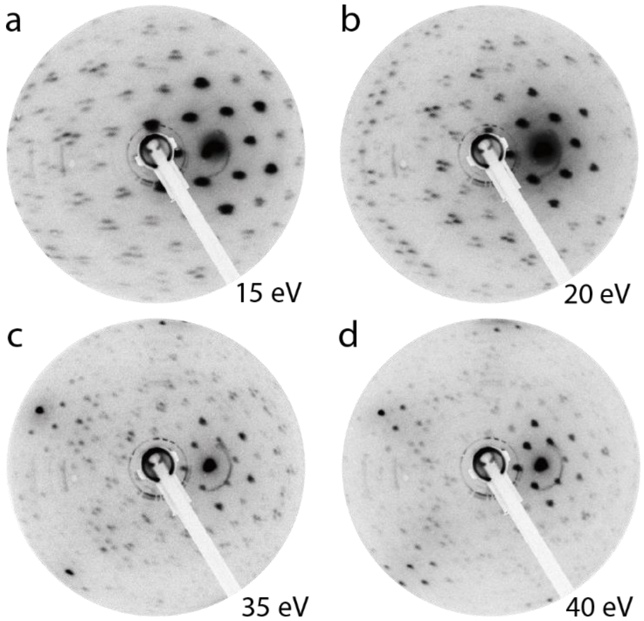

Figure S5. *LEED patterns of Ag-TEB OMN acquired at 90 K with primary electron energy of 15 eV (a), 20 eV (b), 35 eV (c) and 40 eV (d).*

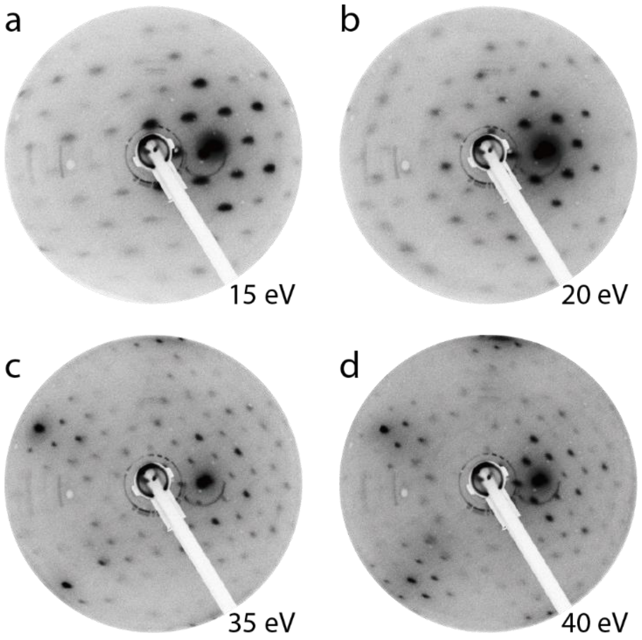

Figure S6. *LEED patterns of Cu-TEB OMN acquired at 90 K with primary electron energy of 15 eV (a), 20 eV (b), 35 eV (c) and 40 eV (d).*

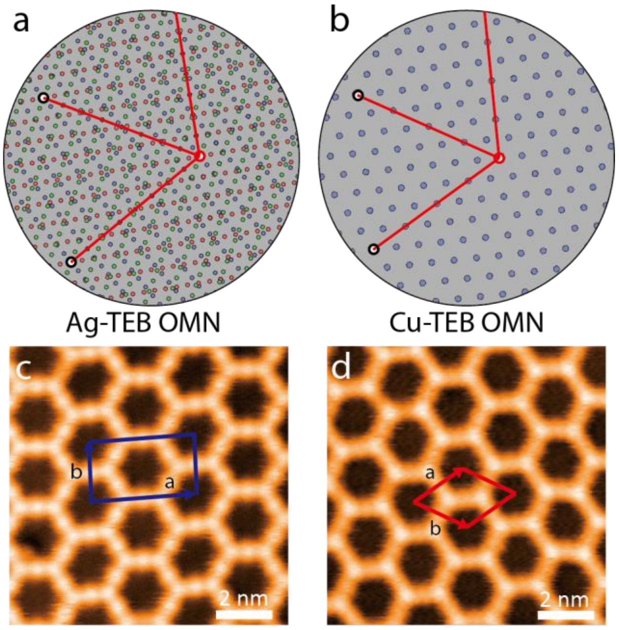

Figure S7. *(a) Simulated LEED pattern for a real-space superstructure matrix* $\begin{pmatrix} 13 & 0 \\ 4 & 8 \end{pmatrix}$ *of Ag-TEB OMN. (b) Simulated LEED pattern corresponding to the real-space superstructure matrix* $\begin{pmatrix} 8 & -4 \\ -4 & 8 \end{pmatrix}$ *for Cu-TEB OMN. (c) STM image of Ag-TEB OMN, with the unit cell marked in blue. (d) STM image of Cu-TEB OMN with red-marked rhombic primitive cell. Tunneling parameters for (c) and (d) are* V_b *= 100 mV,* I_t *= 1 nA.*

Specifically, herein we propose a superstructure matrix $\begin{pmatrix} 13 & 0 \\ 4 & 8 \end{pmatrix}$ for the Ag-TEB OMN periodicity according to models' parameters from Figure S2, as shown in Figure S7a, whose reciprocal-space pattern was simulated via the LEEDpat simulator software[4]. The real-space structure encompasses three inequivalent symmetry domains and the resulting reciprocal-space pattern matches well the experimental LEED pattern of Ag-TEB OMN (Figure 2a and

Figure S5) with characteristic triplet motifs. Additionally, this matrix is consistent with the rectangle unit cell in Figure 1a and Figure S7c with *a* = 37.6 ± 0.2 Å, *b* = 20.7 Å ± 0.2 Å, $\gamma \approx$ 90.2 ± 1°. The agreements emphasize the reasonability of the Ag-TEB OMN model shown in Figure S2. Conversely, the LEED pattern of Cu-TEB OMN shown in Figure S7b can be assigned to the superstructure matrix $\begin{pmatrix} 8 & -4 \\ -4 & 8 \end{pmatrix}$, corresponding to a well-defined $(4\sqrt{3} \times 4\sqrt{3})\mathrm{R}30°$ periodicity relative to the underlying substrate. This assignment is in excellent agreement with the rhombus primitive cell of Figure S7d for Cu-TEB OMN with lattice parameters *a* = 20.1 ± 0.2 Å, *b* = 20.3 ± 0.2 Å, $\gamma \approx$ 59.2º ± 1.0º, and is corroborated by the absence of spot splitting in the LEED patterns of the Cu-TEB OMN at different primary electron energies (cf. Figure S6).

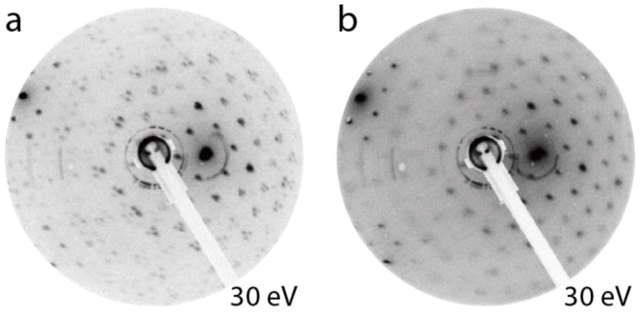

Figure S8*. As-recorded LEED patterns extracted from Figure 2 of the main manuscript without any overlapping drawing. (a) Ag-TEB OMN and (b) Cu-TEB OMN with the same primary electron energy of 30 eV. The disappearance of the triple (split) diffraction spots going from a to b is evident.*

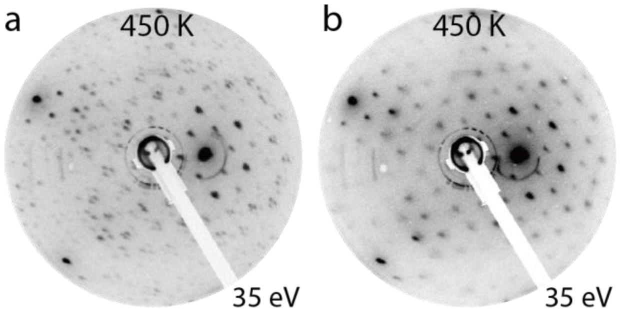

Figure S9*. LEED patterns for (a) Ag-TEB OMN and (b) Cu-TEB OMN after annealing at 450 K, both taken with the same primary electron energy of 35 eV.*

The LEED pattern for Ag-TEB at 450 K is shown in Figure S9a, which maintains the same pattern as Ag-TEB at 375 K (cf. Figure 2a and Figure S8a). The ordered LEED pattern for Ag-TEB could not be obtained at 500 K, since the network decomposed at such temperature. However, the LEED pattern for Cu deposition on Ag-TEB followed by annealing at 450 K is compared in Figure S9b, which shows the same lattice periodicity as Cu-TEB OMN at 500 K (cf. Figure 3b and Figure S8b). The disappearance of triple dot motifs indicating Ag-TEB to Cu-TEB transmetalation can still be recognized clearly.

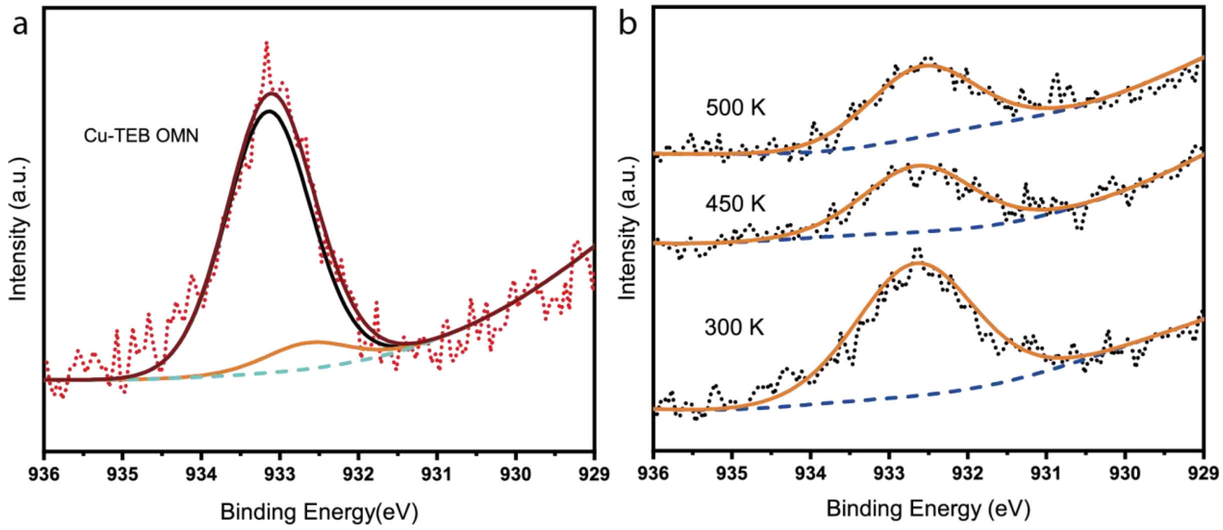

Figure S10. *(a) Peak fit for the XP spectrum of the Cu 2*$p_{3/2}$ *core level of the Cu-TEB OMN after annealing at 500 K. (b) Cu 2*$p_{3/2}$ *XP spectra and fitting analysis for Cu deposited onto the pristine Ag(111) surface after annealing at 300 K (bottom), 450 K (middle) and 500 K (top), respectively.*

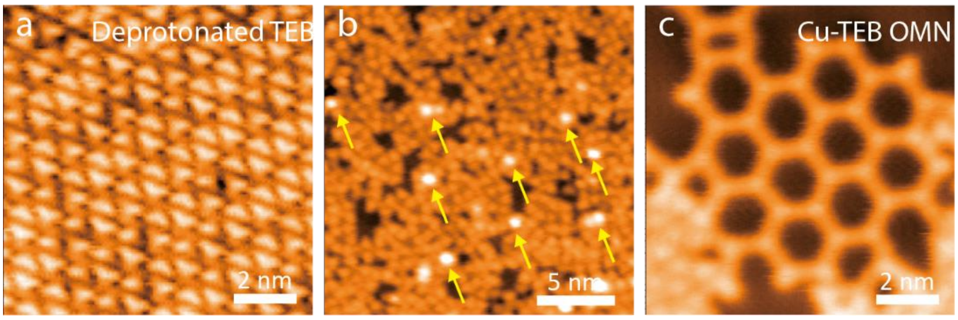

Figure S11. *STM images of (a) deprotonated TEB self-assembly, and of Cu addition onto deprotonated TEB self-assembly at 200 K (b) before and (c) after annealing at 450 K. In (b) Cu clusters are pointed out by yellow arrows. Tunneling parameters: (a)* V_b *= -100 mV,* I_t *= 1 nA; (b)* V_b *= -100 mV,* I_t*= 300 pA; and (c)* V_b *= 100 mV,* I_t*= 1 nA.*

For further study and to complement the transmetalation protocol, we also monitored the preparation of the Cu-TEB OMN under conditions in which the initial formation of Ag-TEB OMN was prevented. Pristine TEB molecules self-assembled on Ag(111) were exposed to O_2 gas at an amount of 600 L at 200 K, forming deprotonated TEB. Subsequently, an appropriate amount of Cu atoms was dispersed onto the deprotonated TEB self-assembly at 200 K, followed by stepwise annealing up to a final temperature of 450 K. In the real-space STM images of Figure S11 the three subsequent stages of this preparation protocol used to form a Cu-TEB OMN are shown. The final structure of panel c matches the Cu-TEB OMN obtained through transmetalation. However, the OMN domains are generally not as large as those by transmetalation, suggesting a more straightforward and effective modification of the Ag-TEB OMN into Cu-TEB OMN.

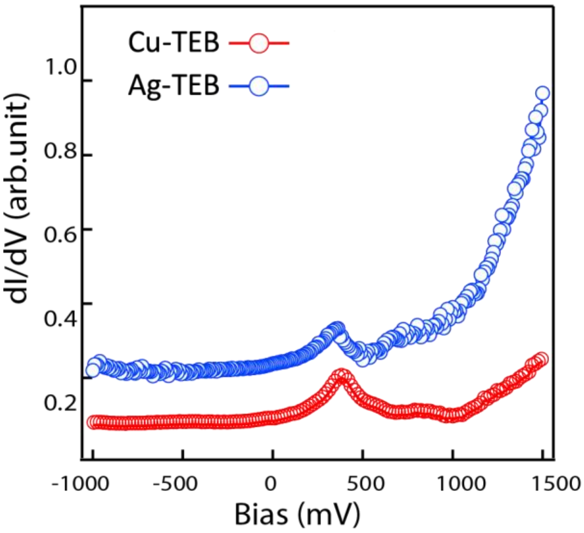

Figure S12. *Long-range dI/dV spectra from -1.0 V to 1.5 V of the Ag-TEB (blue curve) and Cu-TEB (red curve) OMN at the pore centers.*

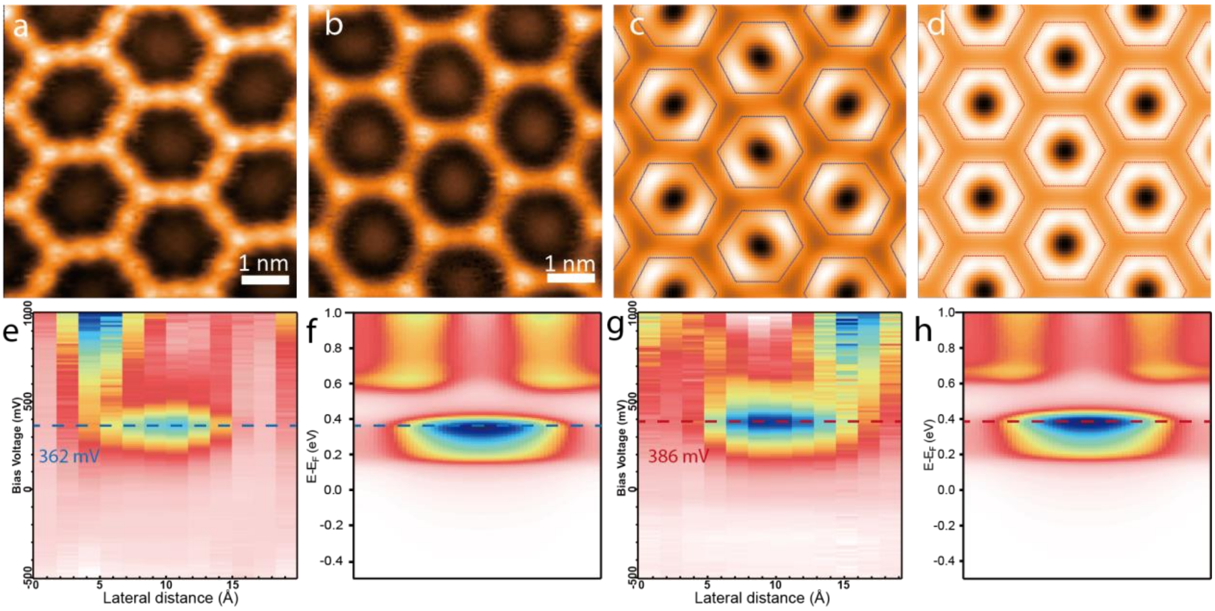

Figure S13. *Constant-height STM images of (a) Ag-TEB OMN at the bias V_b = 360 mV and (b) Cu-TEB OMN at the bias V_b = 380 mV, corresponding to Figure 4b and 4c of the main manuscript, respectively. Theory LDOS map for Ag-TEB at 660 mV (c), and for Cu-TEB OMN at 680 mV (d). Panel (e) and (g) depict STS line spectra across the pore centers of Ag-TEB and Cu-TEB OMN along the white dash line marked in Figure 4b and c. Then simulated LDOS line spectra for Ag-TEB and Cu-TEB OMN are presented in panel (f) and (h), respectively.*

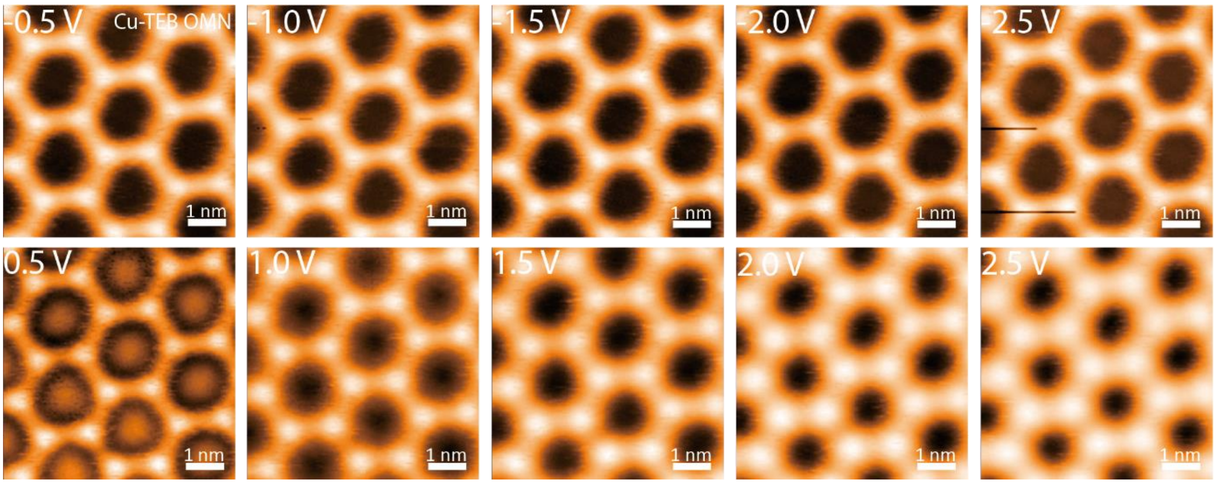

Figure S14. *Bias-dependent STM images for Cu-TEB OMN at $I_t = 300$ pA.*

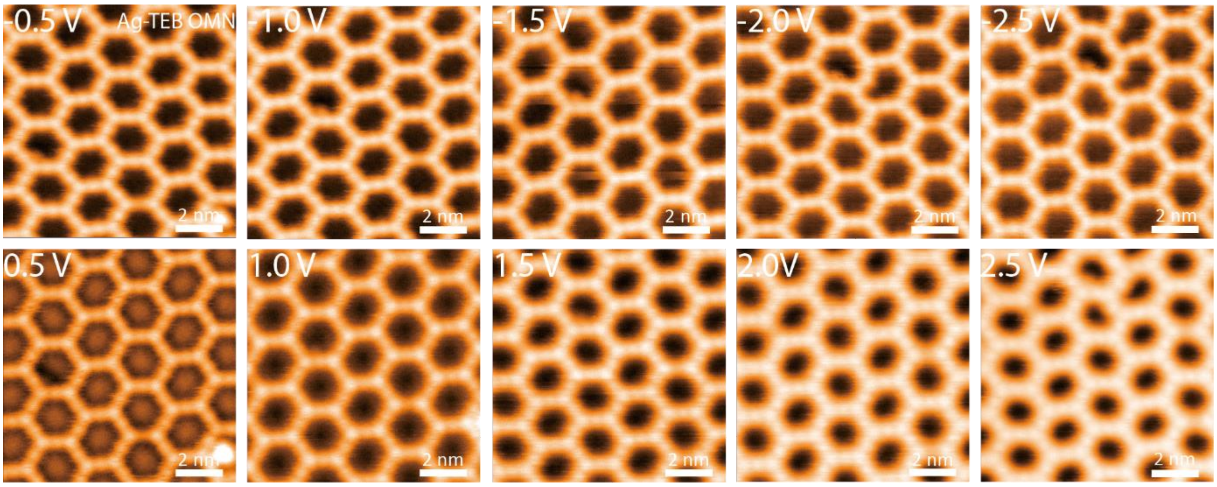

Figure S15. *Bias-dependent STM images for Ag-TEB OMN at $I_t = 1$ nA.*

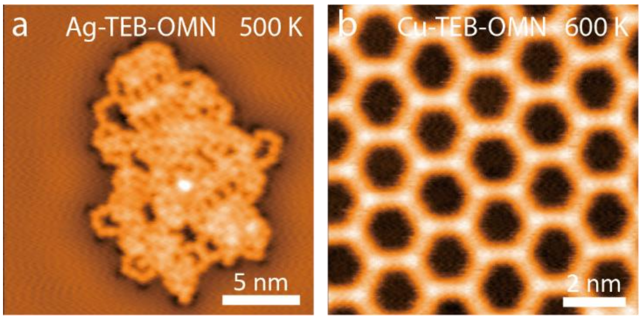

Figure S16. *(a) STM images for the structure of Ag-TEB OMN emerging after annealing at 500 K and for (b) Cu-TEB OMN after annealing at 600 K. Tunneling parameters are $V_b = -100$ mV, $I_t = 500$ pA in (a) and $V_b = -100$ mV, $I_t = 200$ pA in (b).*

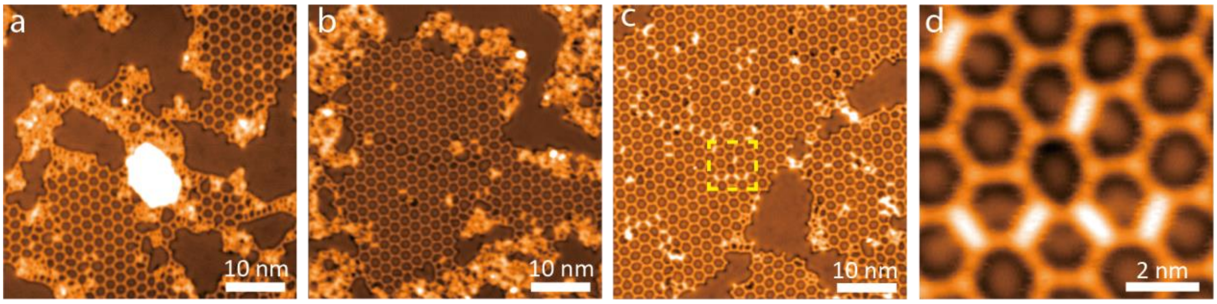

Figure S17. *STM images of Cu-TEB OMN (a) after annealing at 500 K for 1h and (b) at 600 K for 10 mins. (c) Large-area and (d) zoom-in STM images of Cu-TEB OMN containing alkynyl-alkynyl coupling linkages. (a) (c) and (d) were scanned at* V_b *= 380 mV and* I_t *= 300 pA, (b) was scanned at* V_b *= 370 mV and* I_t *= 200 pA,*

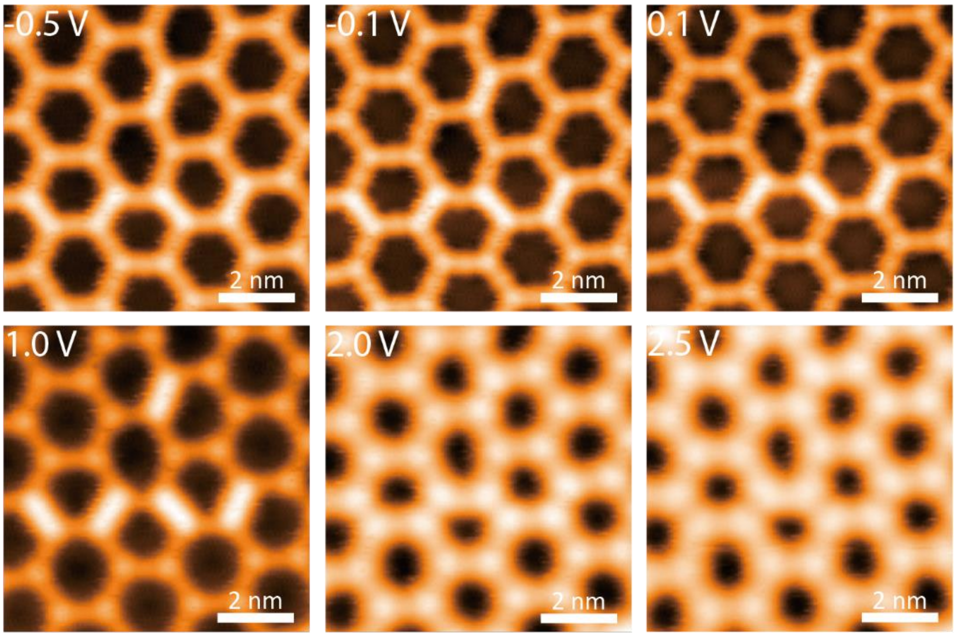

Figure S18. *Bias-dependent STM images of alkynyl coupling structures at* I_t *= 300 pA. The alkynyl-alkynyl linkage has different appearance and contrast with respect to the alkynyl-Cu-alkynyl linkage at 1 V bias, but turns to equal brightness after rising the bias to 2 V.*

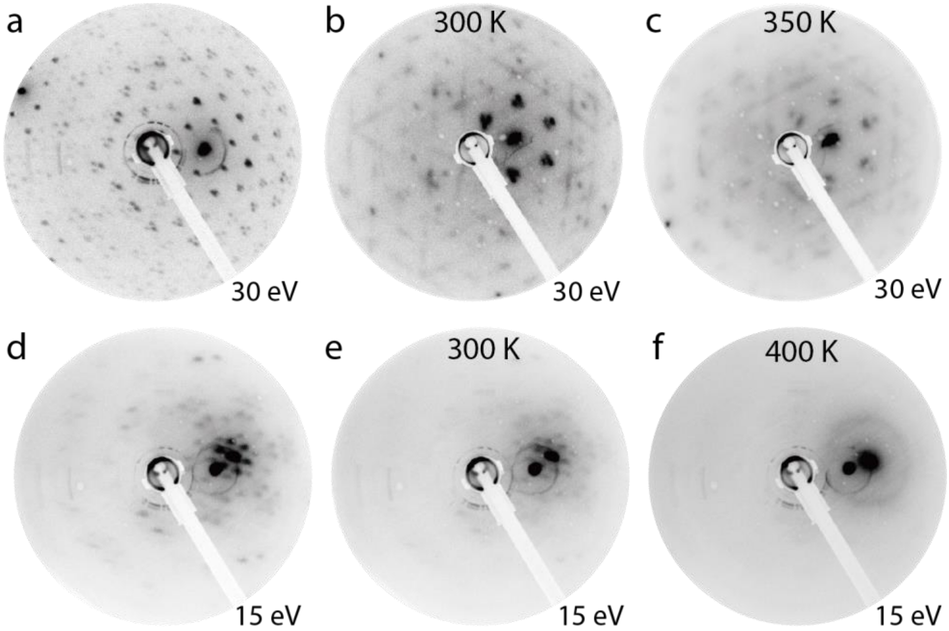

Figure S19*. Top line: LEED patterns for Ag-TEB OMN (a), Bi on Ag-TEB OMN at 300 K (b) and followed by annealing at 350 K (c), acquired at 90 K with primary electron energy of 30 eV. Bottom line: LEED patterns for Ext-TEB OMN (d), Ni deposition on Ext-TEB OMN at 300 K (e) and followed by annealing at 400 K (f), acquired at 90 K with primary electron energy of 15 eV.*

We also tried Bi to replace Ag in the Ag-TEB system by LEED. As shown in Figure S19a, b and c, the addition of Bi still maintains rotational domains leading to some dot triplets reminiscent of the Ag-TEB OMN pattern, but new features indicative of a weaker, new ordered periodic structure appeared, suggesting that Bi may modify the Ag-TEB network. However, the LEED pattern turned into much weaker after annealing at 350 K, proving lower as well as weaker structural order than the Ag-TEB OMN. Therefore, it is unlikely that Bi simply replaces Ag in the networks. Additionally, in the system of Ni combined with the Ext-TEB molecule (which also has terminal alkynyl groups)[2], LEED measurements did not support the successful replacement of Ag with Ni atoms. Figure S19d-f shows the LEED pattern of the Ext-TEB network and its structure worsening upon Ni deposition. in particular, the long-range order is totally disrupted following followed annealing at 400 K (cf. Figure S19f). Thus, these metals could not demonstrate their ability to replace Ag in the pre-existing organometallic system, but instead the observed findings suggest that there may be deeper factors deserving further investigation.

Other used materials: 1,3,5-Tris (4-ethynylphenyl) benzene (Ext-TEB) molecules were synthesized by Svetlana Klyatskaya and Mario Ruben according to a procedure described in Ref [5]. Ni, a highly pure metal foil of 0.2 mm thickness purchased from GoodFellow GmbH, was cut into a ribbon with dimensions of 15 × 20 mm^2 and clamped in between two copper rods. Metallic Bi is typically supplied in the form of flakes and grains from GoodFellow GmbH.

References

(1) Cao, N.; Yang, B.; Riss, A.; Rosen, J.; Björk, J.; Barth, J. V. On-surface synthesis of enetriynes. *Nat. Commun.* **2023,** 14, 1255.

(2) Zhang, Y.-Q.; Paintner, T.; Hellwig, R.; Haag, F.; Allegretti, F.; Feulner, P.; Klyatskaya, S.; Ruben, M.; Seitsonen, A. P.; Barth, J. V.; Klappenberger, F. Synthesizing Highly Regular Single-Layer Alkynyl–Silver Networks at the Micrometer Scale via Gas-Mediated Surface Reaction. *J. Am. Chem. Soc.* **2019,** 141, 5087-5091.

(3) Zhang, Y.-Q.; Paszkiewicz, M.; Du, P.; Zhang, L.; Lin, T.; Chen, Z.; Klyatskaya, S.; Ruben, M.; Seitsonen, A. P.; Barth, J. V.; Klappenberger, F. Complex supramolecular interfacial tessellation through convergent multi-step reaction of a dissymmetric simple organic precursor. *Nat. Chem.* **2018**, 10, 296-304.

(4) Hermann, K.; Van Hove, M. A. Software Utility LEEDpat (Version 4.2); Fritz Haber Institute: https://www.fhi.mpg.de/958975/LEEDpat4
(5) Kepčija, N.; Zhang, Y.-Q.; Kleinschrodt, M.; Björk, J.; Klyatskaya, S.; Klappenberger, F.; Ruben, M.; Barth, J. V. Steering On-Surface Self-Assembly of High-Quality Hydrocarbon Networks with Terminal Alkynes. *J. Phys. Chem.C.* **2013**, 117, 3987-3995.
